# The Trw Type IV Secretion System of *Bartonella* Mediates Host-Specific Adhesion to Erythrocytes

**DOI:** 10.1371/journal.ppat.1000946

**Published:** 2010-06-10

**Authors:** Muriel Vayssier-Taussat, Danielle Le Rhun, Hong Kuan Deng, Francis Biville, Sandra Cescau, Antoine Danchin, Geneviève Marignac, Evelyne Lenaour, Henri Jean Boulouis, Maria Mavris, Lionel Arnaud, Huanming Yang, Jing Wang, Maxime Quebatte, Philipp Engel, Henri Saenz, Christoph Dehio

**Affiliations:** 1 Unité Sous Contrat Bartonella, INRA, Maisons-Alfort, France; 2 Focal Area Infection Biology, Biozentrum, University of Basel, Basel, Switzerland; 3 UMR BIPAR, Equipe des Bactéries Zoonotiques Hémotropes, ENVA/AFSSA, Maisons-Alfort, France; 4 Unité des Génomes Bactériens, Institut Pasteur, CNRS URA 2171, Paris, France; 5 AMAbiotics, Evry, France; 6 Institut de Transfusion Sanguine, Paris, France; 7 Beijing Genomics Institute, Shenzhen, China; 8 Institute of Psychology, Chinese Academy of Sciences, Beijing, China; Yale University School of Medicine, United States of America

## Abstract

Bacterial pathogens typically infect only a limited range of hosts; however, the genetic mechanisms governing host-specificity are poorly understood. The α-proteobacterial genus *Bartonella* comprises 21 species that cause host-specific intraerythrocytic bacteremia as hallmark of infection in their respective mammalian reservoirs, including the human-specific pathogens *Bartonella quintana* and *Bartonella bacilliformis* that cause trench fever and Oroya fever, respectively. Here, we have identified bacterial factors that mediate host-specific erythrocyte colonization in the mammalian reservoirs. Using mouse-specific *Bartonella birtlesii*, human-specific *Bartonella quintana*, cat-specific *Bartonella henselae* and rat-specific *Bartonella tribocorum*, we established *in vitro* adhesion and invasion assays with isolated erythrocytes that fully reproduce the host-specificity of erythrocyte infection as observed *in vivo*. By signature-tagged mutagenesis of *B. birtlesii* and mutant selection in a mouse infection model we identified mutants impaired in establishing intraerythrocytic bacteremia. Among 45 abacteremic mutants, five failed to adhere to and invade mouse erythrocytes *in vitro*. The corresponding genes encode components of the type IV secretion system (T4SS) Trw, demonstrating that this virulence factor laterally acquired by the *Bartonella* lineage is directly involved in adherence to erythrocytes. Strikingly, ectopic expression of Trw of rat-specific *B. tribocorum* in cat-specific *B. henselae* or human-specific *B. quintana* expanded their host range for erythrocyte infection to rat, demonstrating that Trw mediates host-specific erythrocyte infection. A molecular evolutionary analysis of the *trw* locus further indicated that the variable, surface-located TrwL and TrwJ might represent the T4SS components that determine host-specificity of erythrocyte parasitism. In conclusion, we show that the laterally acquired Trw T4SS diversified in the *Bartonella* lineage to facilitate host-restricted adhesion to erythrocytes in a wide range of mammals.

## Author Summary

Pathogens are—as the result of adaptive evolution in their principal host(s)—typically limited in the range of hosts that they can infect successfully. However, infrequently such host-restricted pathogens may undergo a spontaneous host switch, which can lead to the evolution of pathogens with altered host specificity. Most human pathogens evolved this way, and animal-specific pathogens have thus to be considered as an important reservoir for the emergence of novel human pathogens. Despite host-specificity representing a common feature of pathogens, the underlying molecular mechanisms are largely unknown. In this study we have used bacterial pathogens of the genus *Bartonella* to identify bacterial factors involved in the determination of host specificity. The bartonellae represent an excellent model to study host-specificity as each species is adapted to cause an intracellular infection of erythrocytes exclusively in its respective reservoir host(s). Using a genetic approach in combination with erythrocyte infection models *in vitro* and *in vivo* we demonstrate that a surface-located bacterial nanomachine—a so-called type IV secretion system—determines host specificity of erythrocyte infection. Our work sheds light on the molecular basis of host specificity and establishes an experimental model for studying the evolutionary processes facilitating spontaneous host shifts.

## Introduction

The successful infection of a mammalian host by a bacterial pathogen typically involves a series of intimate host-pathogen interactions. On the molecular level this is reflected by specific receptor-ligand interactions between bacterial virulence factors and their targeted host factors [1]. Adaptation of a bacterial virulence factor to a host factor that displays variability within the host population can restrict the host range that is susceptible to infection. The resulting host-specificity is an inherent feature of most bacterial pathogens of humans, including *Helicobacter pylori*, *Listeria monocytogenes, Neisseria gonorrhoae*, *Salmonella typhi*, *Streptococcus pyogenes* and *Staphylococcus aureus*. However, remarkably little is known about the molecular determinants of host specificity in bacterial infections, with the only exception of *L. monocytogenes* for which the conjugated action of two distinct host-specific invasion proteins was shown to be critical for fetoplacental listeriosis [2], [3], [4].

Bartonellae represent an interesting but largely unexplored model for host specificity. These facultative intracellular bacteria use arthropod transmission and hemotropism as mammalian parasitism strategies [5]. As the result of an adaptive radiation each of the 21 species infects only one or a few closely related mammalian reservoir host(s), which is highlighted by their capacity to cause a long-lasting intraerythrocytic bacteremia [6]. Non-reservoir hosts may get incidentally infected without developing an intraerythrocytic infection [7]. Two *Bartonella* species are human-specific: *Bartonella bacilliformis* causes the biphasic Carrion's disease, with acute Oroya fever followed by the chronic verruga peruana, and *Bartonella quintana* causing trench fever. The life-threatening Oroya fever and the much milder course of trench fever represent the characteristic intraerythrocytic stages of these pathogens. The other 19 species cause intraerythrocytic infections in various non-primate mammalian reservoirs. At least seven of them are recognized as zoonotic pathogens which incidentally infect humans. Commonly, *B. henselae* is associated with cat scratch disease [7].

The life cycle of *Bartonella* in the reservoir host has been analyzed in detail in rats experimentally infected with *B. tribocorum* [8]. Following intravenous inoculation, bacteria initially infect a primary niche outside of circulating blood, which is considered to comprise the vascular endothelium and possibly other cell types. Approximately on day five of infection, large numbers of bacteria are released into the bloodstream where they bind to and invade mature erythrocytes. Bacteria then replicate in a membrane-bound compartment until reaching a critical number. For the remaining life span of the erythrocytes the intracellular bacteria remain in a non-dividing state [8]. Monitoring of bacteremia in other animal models, such as the *B. birtlesii*-mouse [9] and *B. henselae*-cat models [10], or in captive naturally infected animals has yielded results that match those observed in the *B. tribocorum*-rat model, suggesting a common mode of infection of the different species in their respective animal reservoirs [11]. The only exception is *B. bacilliformis*, which causes lysis of the infected human erythrocytes, eventually resulting in a severe hemolytic anemia.

The *B. tribocorum*-rat model was further explored to identify bacterial pathogenicity factors that are required for colonization of the mammalian reservoir host. A signature-tagged mutagenesis (STM) screen identified 98 essential bacterial loci [12], including genes encoding components of two distinct type IV secretion systems (T4SS), VirB/VirD4 and Trw, the invasion-associated locus B (IalB) protein, the trimeric autotransporter adhesin BadA, as well as further members of the autotransporter family [6]. Whether any of the identified genes is critical for host-specificity is unknown, although it is conceivable to assume that host-specificity loci are essential for infection and may thus be represented among the hits of the performed STM screen.

Experimental infections of different mammalian hosts by a given *Bartonella* strain have reproduced the species-specificity of erythrocyte invasion as observed in natural infections [11], [13], [14], [15]. However, despite their availability, *in vitro* erythrocyte infection assays [16], [17] have not been investigated for the study of host specificity. Here, we demonstrate for the first time that host specificity is reflected by the exclusive capacity of *Bartonella* species to adhere to erythrocytes isolated from their natural host(s). Second, by performing STM in *Bartonella birtlesii* followed by screening in mice *in vivo* and in isolated erythrocytes *in vitro* we identified the T4SS Trw as the molecular determinant of host-specific erythrocyte infection.

## Results

### An *in vitro* erythrocyte colonization assay to study host-restricted infection

**Figure 1:**
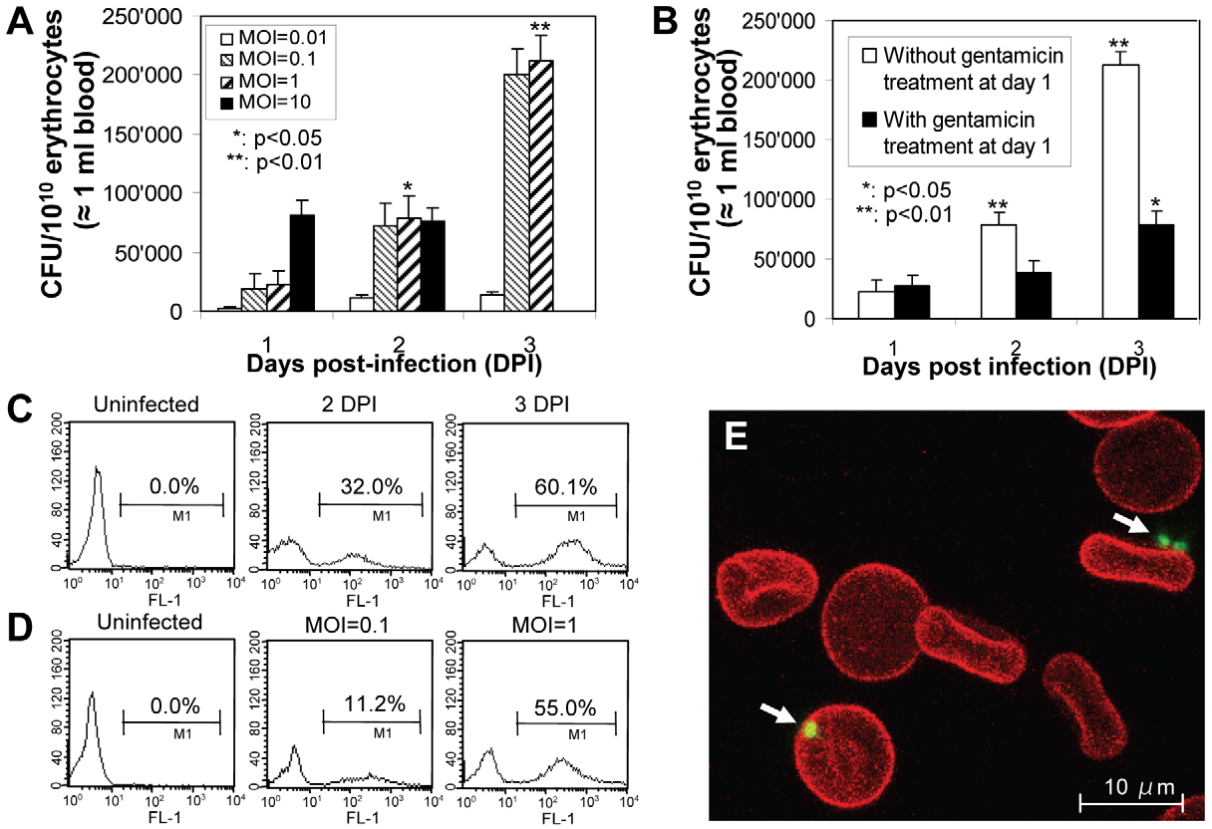
*B. birtlesii* invades murine erythrocytes *in vitro*. (**A**, **B**) Time- and bacterial number-dependency of *B. birtlesii* invasion of murine erythrocytes determined by the gentamicin protection assay. (**A**) Freshly isolated murine erythrocytes were infected with *B. birtlesii* at the indicated multiplicity of infection (MOI) and the numbers of intra-erythrocytic bacteria (colony forming units, CFU) was determined by the gentamicin protection assay at 1, 2 and 3 days post infection (DPI); n = 6, mean +/-SD; *, **: significant difference of data compared to 1 DPI. (**B**) Freshly isolated murine erythrocytes were infected with *B. birtlesii* at MOI = 1. At 1 DPI, gentamicin was added to half of the samples, and growth was continued in the continous presence of gentamicin through 3 DPI to kill extracellular bacteria. The other half of the infected erythrcyotes was not treated with gentamicin. For both untreated and gentamicin treated samples, numbers of intra-erythrocytic bacteria were determined by the gentamicin protection assay at 1, 2 and 3 DPI (n = 6; mean +/−SD, *, **: significant difference in gentamicin treated samples compared to 1 DPI). (**C**) Time- and (**D**) bacterial number-dependency of *B. birtlesii* associated to murine erythrocytes determined by flow cytometry. Freshly isolated murine erythrocytes were infected with *B. birtlesii-gfp* (MOI = 1, detection at 1 and 3 DPI in C and MOI = 0.1 or 1, detection at 3 DPI in D). The percentage of erythrocytes associated with bacteria were quantified by flow cytometric analysis at 2 and 3 DPI. Representative data for the fluorescence (FL-1) of 10′000 erythrocytes are shown as histogram plots. (**E**) Confocal microscopic analysis of murine erythrocytes infected for 2 days with GFP-expressing *B. birtlesii* (MOI = 1). Arrows point to bacteria found in close association with erythrocytes.

Based on described *in vitro* models of human and feline erythrocyte infection by *B. bacilliformis* and *B. henselae*, respectively [16], [17], we established for *B. birtlesii* an *in vitro* infection model for erythrocytes isolated from the murine reservoir host. Balb/C mice were used as the source of erythrocytes as they are known to develop a long lasting intraerythrocytic infection upon experimental infection with *B. birtlesii* [9]. The intraerythrocytic presence of bacteria was evaluated over a period of three days using the gentamicin protection assay (Fig. 1A). Bacterial entry into erythrocytes was dependent on the number of bacteria per erythrocyte (multiplicity of infection, MOI; tested MOI range: 0.01 to 10) and time of infection (days post infection, DPI; tested time range: 1 to 3 DPI). The highest intraerythrocytic bacterial content over time was obtained for MOI = 0.1 and 1, with approximately 2×10^5^ colony forming units (CFU) per 10^10^ erythrocytes (≈0.002% infected erythrocytes) at 3 DPI. Given that mouse blood contains approximately 10^10^ erythrocytes/ml, this value corresponds well to the bacteremia reported for experimentally infected Balb/C mice (≈1×10^5^ CFU/ml; 0.001% infected erythrocytes) [9]. For MOI = 10, erythrocytes were infected at 1 DPI, but lysed entirely by 3 DPI. At MOI = 0.01, only low numbers of intraerythrocytic bacteria were detected over time. Based on these data, MOI = 1 was used for all subsequent erythrocyte infection assays. To evaluate whether the increase of intraerythrocytic bacteria over time was mainly due to continued bacterial invasion, or to intraerythrocytic bacterial multiplication, or to a combination of both, erythrocytes were infected with *B. birtlesii* for one day in the absence of gentamicin, followed by incubation in the continuous presence of gentamicin to kill extracellular bacteria. Fig. 1B shows that the number of intracellular bacteria increased over time in the presence of gentamicin, albeit to a lesser extent than in the untreated control. Bacteria thus appear to enter erythrocytes beyond 1 DPI and, moreover, to replicate in an intra-erythrocytic location.

Invasion of erythrocytes by *Bartonella* is preceded by bacterial adhesion to the erythrocyte surface [18]. To quantify erythrocytes infected by adherent extracellular and/or intracellular bacteria, we used GFP-expressing bacteria in combination with flow cytometry (Fig. 1C, D.). Similar as described for intraerythrocytic bacteria in the gentamicin protection assay, erythrocyte colonization revealed by flow cytometry was dependent on time (Fig. 1.C) and MOI (Fig. 1.D). However, the rate of erythrocyte colonization evaluated by flow cytometry (55% for MOI = 1 at 3 DPI) was approximately 20′000-fold higher than erythrocytes invasion determined by the gentamicin protection assay (compare Fig. 1.A), indicating that the vast majority of bacteria detected by flow cytometry were associated extracellularly with erythrocytes. Confocal microscopy confirmed the predominant extracellular localization of erythrocyte-associated bacteria (Fig. 1.E).

**Figure 2:**
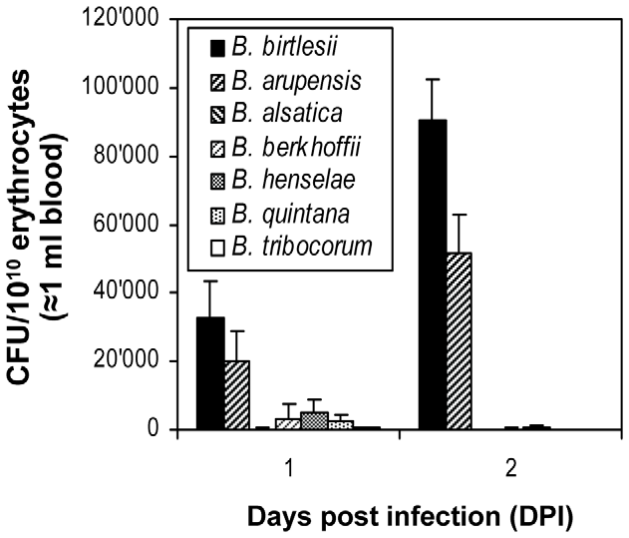
Efficiency of *in vitro* invasion of murine erythrocytes by different *Bartonella* species. Freshly isolated murine erythrocytes were infected with the indicated *Bartonella* species with a MOI = 1. The numbers of intra-erythrocytic bacteria (colony forming units, CFU) was determined by the gentamicin protection assay at 1 and 2 days post infection (DPI); mean +/-SD of triplicate samples.

**Figure 3:**
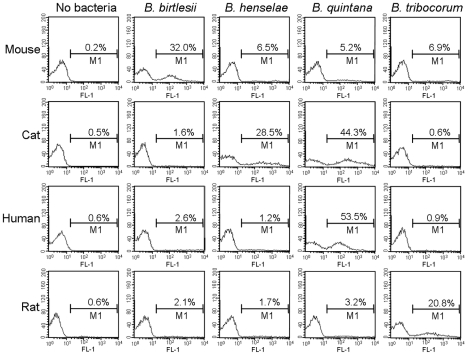
Efficiency of interaction between erythrocyte and *Bartonella* sp. according to host origin and *Bartonella* species. Freshly isolated erythrocytes from mouse, cat, human or rat were infected with *gfp*-expressing bacteria of the indicated *Bartonella* species (MOI = 1). The percentages of infected erythrocytes were determined by flow cytometry at two DPI. Representative histogram plots for GFP-fluorescence (FL-1) of 10′000 erythrocytes are shown.

Next we investigated whether *Bartonella* species differ in their capacity to interact *in vitro* with erythrocytes of different mammalian origin, and whether this capacity may reflect the host-restriction displayed during natural infection. First, mouse erythrocytes were infected with either *B. birtlesii*, *B. vinsonii arupensis* (both mouse-specific), *B. alsatica* (rabbit-specific), *B. vinsonii berkhoffii* (dog-specific), *B. henselae* (cat-specific), *B. quintana* (human-specific), or *B. tribocorum* (rat-specific). Erythrocyte invasion was quantified by the gentamicin protection assay (Fig. 2). *B. vinsonii arupensis* displayed invasion rates similar to *B. birtlesii*, while none of the other strains tested resulted in significant erythrocyte invasion. Using a corresponding set of strains expressing GFP, consistent results were obtained for the flow cytometric determination of bacterial adhesion to mouse erythrocytes (Fig. 3 and Fig. S1). These findings indicate that specificity for the mouse reservoir *in vivo* correlates with efficient adhesion to and invasion of mouse erythrocytes *in vitro*.

Next, we tested whether - similarly as observed for mouse erythrocytes and *B*. *birtlesii* – the capacity of *B. henselae, B. quintana* and *B. tribocorum* to adhere to erythrocytes *in vitro* is also restricted to erythrocytes from their natural reservoir host, i.e. cat, human and rat, respectively. GFP-expressing bacteria were used for erythrocyte infection, and adhesion was quantified by flow cytometric analysis. Fig. 3 and Table 1 illustrate that all tested *Bartonella* species were able to efficiently adhere to erythrocytes isolated from their respective reservoir hosts, while they essentially did not adhere to erythrocytes from non-reservoir hosts. The only exception is *B. quintana*, which further to erythrocytes from the human reservoir also colonized cat erythrocytes. Together, these data indicate that the established *in vitro* model of erythrocyte colonization reflects well the host restriction as observed during natural infection.

**Table 1:** Efficiency of erythrocyte colonization according to host origin and *Bartonella* species.

	*B. birtlesii*		*B. henselae*		*B. quintana*		*B. tribocorum*	
	*in vivo*	*in vitro* (%)*	*in vivo*	*in vitro* (%)*	*in vivo*	*in vitro* (%)*	*in vivo*	*in vitro* (%)*
**Mouse**	+^a^	26.3 +/− 2.2	-b	1.3 +/− 0.5	n.r.	0.8 +/− 0.3	n.r.	1.6 +/− 0.4
**Cat**	n.r.	1.6 +/− 0.8	+^c^	28.5 +/− 4.1	n.r.	42.2 +/− 3.1	n.r.	0.5 +/− 0.3
**Human**	n.r.	2.7 +/− 1.0	n.r.	1.3 +/− 0.3	+^d^	58.4 +/− 1.2	n.r.	0.9 +/− 0.2
**Rat**	n.r.	2.0 +/− 1.3	n.r.	1.8 +/− 0.7	n.r.	3.5 +/− 1.1	+^e^	20.7 +/− 2.8

^a^ [9],

^b^ [15],

^c^ [41], [42],

^d^ [8], [43], [44],

^e^ [8].

*Freshly isolated erythrocytes from mouse, cat, human or rat were infected with *gfp*-expressing bacteria of the indicated *Bartonella* species (MOI = 1). The percentages of colonized erythrocytes were determined by flow cytometry at day two post infection. Data for 10′000 erythrocytes per time-point were analyzed (n = 6 for tests with homologous species and n = 3 for tests with heterologous species, mean +/− SD). For previously described infections of the respective mammalian hosts with the indicated *Bartonella* species the presence (+) or absence (-) of intraerythrocytic bacteremia is indicated (n.r. = not reported).

### *B. birtlesii* genes required for intra-erythrocytic bacteremia in mice

As a basis for identifying genetic factors involved in host-restricted erythrocyte colonization, we identified a comprehensive set of *B. birtlesii* genes required for establishing intraerythrocytic bacteremia in mice. To this end, an STM library of *B. birtlesii* was constructed as previously described for *B. tribocorum* [6], [12], [19]. From each conjugation assay, we selected 96 single kanamycin-resistant colonies and assembled an STM mutant library of 3456 mutants. We then identified mutants that have lost the capacity to cause intraerythrocytic bacteremia by screening the library in the mouse infection model [9]. Of 1456 mutants tested in the input pools, 98 were not detected in the output pools from mice at days 7 and 14 post infection and were thus classified as abacteremic mutant candidates. All 98 abacteremic mutant candidates were retested by reassembling them into 49 pools of 9 mutants, each pool containing two abacteremic mutant candidates and an invariable set of seven mutants displaying wild-type behavior (bacteremic mutants). The rescreen confirmed an abacteremic phenotype for 48 of the initial 98 abacteremic mutant candidates, corresponding to 3.3% of the total number of mutants screened. Growth of all of the 48 confirmed abacteremic mutants on solid media was similar to the parental wild-type strain (data not shown).

We determined the transposon insertion sites for all 48 abacteremic mutants by direct sequencing out of the transposon into the flanking chromosomal region and mapping of the derived sequences onto the draft genome sequence of *B. birtlesii* (S. Cescau, H.M. Yang, J. Wang, M. Vayssier-Taussat, A. Danchin, and F. Biville, unpublished data). Three mutants harboring two separate transposon insertions were not considered further in the analysis. *Table S1* lists the loci inactivated by single transposon insertion in the remaining 45 abacteremic mutants. Five mutants carried the transposon insertion in an intergenic region: one (83D04) was near a gene encoding a tRNA; three of them (04A01, 86C05, 69B07) were upstream of genes encoding proteins of unknown function and one (69C09) was in proximity to a putative transcriptional regulator gene. In these mutants, the transposon may have thus disrupted a promoter or another regulatory sequence. 40 transposon insertions were mapped to the coding region of 38 different protein-encoding genes. In 8 mutants the insertions were found in genes encoding a conserved protein of unknown function, among them three putative surface proteins. Sixteen mutants carried insertions in genes previously implicated in bacterial pathogenicity, either in *Bartonella* (*virB/D4*, *trw*, *ialA/B*, *badA*, *omp43*, *iba*) or other pathogenic bacteria (i.e. loci encoding heat shock proteins) [5]. Moreover, mutant genes encoding proteins involved in transport and metabolism, as well as phage-related function were also identified.

### *B. birtlesii* genes required for erythrocytic infection *in vitro*

**Figure 4:**
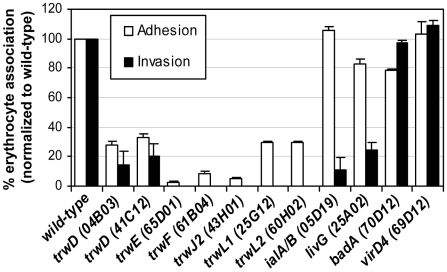
Role of Trw in erythrocyte infection. Efficiency of *in vitro* invasion of murine erythrocyte by abacteremic mutants of *B. birtlesii*. The *in vitro* erythrocyte adhesion or invasion phenotype of abacteremic mutants identified in the STM screen was evaluated at 2 DPI by flow cytometry after immunocytochemical staining of bacteria (see Figure S2) or at 1 DPI by the gentamicin protection assay, respectively. The efficiency of erythrocyte adhesion or invasion of each tested mutant is expressed as percentage of erythrocyte adhesion or invasion of the isogenic wild-type strain (mean +/- SD of triplicate samples). All mutants listed in *Table S1* that do not appear in this figure display wild-type phenotype in regard of *in vitro* erythrocyte invasion.

The 45 confirmed abacteremic mutants with single transposon insertion were individually tested for their capacity to invade murine erythrocytes using the gentamicin protection assay (*Table S1*). Nine mutants were found to be impaired in murine erythrocyte invasion (Fig. 4). Complementary erythrocyte adhesion assays based on flow cytometric analysis of antibody-stained bacteria demonstrated that seven of these nine invasion-deficient mutants are also deficient in erythrocytes adhesion. All seven mutants harbor a mutation in the operon encoding the T4SS Trw (two in *trwD*, *trwE*, *trwF, trwJ2, trwL1, trwL2)*, which was previously shown to be important for establishing an intraerythrocytic bacteremia in *B. tribocorum* [20]. Compared to wild-type, both *trwD* mutants (04B03 and 41C12) showed a five-fold decrease in invasion/adhesion efficiency. All other *trw* mutants failed to invade erythrocytes and were severely impaired in their capacity to adhere to erythrocytes (Fig. 4). These data demonstrate that the Trw system is required for erythrocyte invasion by mediating specific adhesion to erythrocytes. In contrast, mutants harboring an insertion in the invasion-associated locus *ialA/B* showed normal erythrocyte adhesion but impaired invasion (10-fold reduced, p<0.01), confirming the previously suggested role of this locus in erythrocyte invasion [21], [22]. Equally, an insertion mutant (25A02) inactivating *livG* (encoding an amino acid ABC-transporter) showed normal adhesion but a specific defect in invasion (4-fold, p<0.05) compared to wild-type (Fig. 4). None of the other abacteremic mutants appeared to be involved in erythrocytes invasion indicating that they probably are required for an earlier step of infection, i.e. for colonization of the primary niche.

### Role of Trw T4SS in host-specific infection of erythrocytes

**Figure 5:**
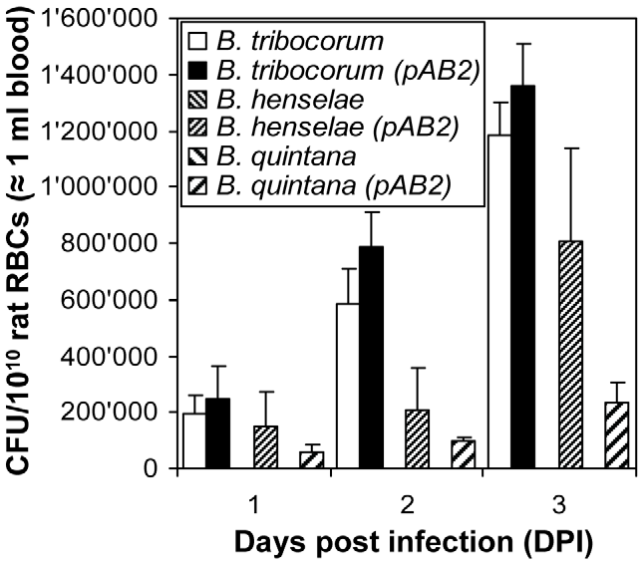
Role of Trw T4SS in mediating host-specific erythrocyte invasion. Freshly isolated rat erythrocytes were infected with *B. tribocorum, B. tribocorum* (pAB2), *B. henselae*, *B. henselae* (pAB2), *B. quintana*, or *B. quintana* (pAB2) at a MOI = 1. Intra-erythrocytic bacteria were enumerated at 1, 2 and 3 days post infection (DPI) by the gentamicin protection assay (n = 3; mean +/−SD).

Next we tested whether the Trw system shown here to be essential for erythrocyte infection *in vitro* and *in vivo* may be directly involved in determining host-specificity. To this end we introduced pAB2, a plasmid encoding the *trw* locus of rat-specific *B. tribocorum* [20], into cat-specific *B. henselae*, human-specific *B. quintana* and *B. tribocorum* (control). We then compared the capacity of these recombinant strains to infect rat erythrocytes with their parental strains. Among the parental strains, only *B. tribocorum* mediated invasion of rat erythrocytes (Fig. 5), which is consistent with the erythrocyte adhesion data presented in Fig. 2. The pAB2-mediated ectopic over-expression of *trw* in *B. tribocorum* resulted only in a slight increase of invasion, indicating that the endogenous level of trw expression is sufficient to mediate efficient bacterial entry. Strikingly, ectopic expression of the *B. tribocorum trw* locus in *B. henselae* and *B. quintana* rendered these pathogens capable of infecting rat erythrocytes. These data clearly demonstrate a direct role of the Trw system in determining host-specificity of erythrocyte infection.

To further assess which components of the Trw T4SS may mediate host specificity we analyzed the molecular evolution of different *trw* genes of *B. birtlesii* and related species. Candidate genes for mediating host specificity are surface exposed components, i.e. the T4SS pilus components TrwL and TrwJ. As shown by Nystedt *et al.* [23] for other *Bartonella* species, *trwL* and *trwJ* genes have been amplified and diversified several times during evolution. The *trw* locus of *B. birtlesii* also displays amplification of *trwL* (five copies) and co-amplification of *trwJ* together with *trwH and trwI* (two copies) (Fig. S3, panel A). Phylogenetic analyses and calculation of the non-synonymous (*d*N) and synonymous (*d*S) substitution frequencies of different *trw* genes further showed that *trwJ* and *trwL* homologs have diversified to much higher degree than other components of the Trw T4SS, within and among different species [Fig. S3, panel B-G, and [23]].

## Discussion

Host-specificity is a prominent feature of pathogenic bacteria that reflects the host range susceptible to infection. Subtle changes in the molecular mechanisms that govern host-specificity may result in spontaneous host shifts, which represent a major risk for the emergence of novel human pathogens from animal reservoirs. Striking examples for this evolutionary scenario are the bartonellae, which cause host-restricted intra-erythrocytic infections in their mammalian reservoirs. In conjunction with repeated host shifts, the large number of *Bartonella* species evolved by adaptive radiation [6], including the human-specific pathogen *B. quintana* that evolved from cat-specific *B. henselae* [24]. Here we explored the bacterial genetic basis for host-restricted infection of erythrocytes. The establishment of an *in vitro* model of erythrocyte adherence and invasion allowed us to demonstrate for the first time a direct correlation of host-restricted erythrocyte infection *in vivo* and *in vitro*, demonstrating that host-specificity is determined by the capacity of bacteria to adhere to erythrocytes. In order to identify the bacterial factors critical for host-restricted erythrocyte infection we have used a two-step experimental protocol. First, we performed an STM screen for *B. birtlesii* in mice which allowed us to identify 45 abacteremic mutants defective in establishing intra-erythrocytic infection. Among the corresponding set of 38 protein-encoding genes, 13 loci were also indentified in a similar STM screen performed in the *B. tribocorum*-rat model [6]. This indicates extensive similarities in the repertoire of pathogenesis factors in these closely related organisms as well as robustness of the performed genetic screens. Second, rescreening of the entire set of 45 abacteremic *B. birtlesii* mutants in the *in vitro* mouse erythrocyte infection model resulted in the identification of nine mutants impaired in erythrocte invasion. The other mutants (36 of 45 = 80%) displaying a wild-type phenotype in this assay are therefore not directly involved in erythrocyte infection, but rather may contribute to the establishment of infection in the primary niche. Prominent examples are the *virB/virD4* genes encoding the VirB/VirD4 T4SS, which is known to be required for primary niche infection in the *B. tribocorum*/rat model (Schulein, 2002). Moreover, a recent study inferred the VirB/VirD4 T4SS as major bacterial factor facilitating bacterial adaptation to novel hosts [6]. The nine mutants impaired in *in vitro* erythrocyte invasion differ in their capacity to adhere to erythrocytes. Transposon insertions in the invasion locus (*ialA/B*) previously implicated in erythrocyte invasion [21], [22] and *livG* encoding an amino acid ABC-transporter displayed wild-type like adherence to erythrocytes. IalA/B and LivG should thus represent invasion factors. The remaining seven invasion-deficient transposon mutants were all severely impaired in erythrocyte adhesion. Strikingly, all these mutants carry insertions in components encoding the T4SS Trw, which thus represents an erythrocyte adherence system that is critical for erythrocyte invasion. Trw is known to be required for establishing intra-erythrocytic infection in the *B. tribocorum-*rat model [5], [20], [25], however, evidence for a direct role of the Trw system in erythrocyte adhesion as provided here was lacking so far. Based on the presumable surface location of components of Trw [20] this T4SS may directly interact with the erythrocyte surface and thus may restrict the host range of erythrocyte infection. To test the hypothesis that Trw determines host range we have expressed Trw of rat-specific *B. tribocorum* in cat-specific *B. henselae* and human-specific *B. quintana*. Strikingly, this genetic manipulation resulted in an extension of the host range for *in vitro* erythrocyte infection towards rats, demonstrating that Trw indeed represents a major determinant of host-specificity of erythrocyte infection. Thus, this finding establishes a new experimental model to study the molecular mechanisms governing host restriction - further to the molecular paradigm of host-specificity exemplified by the interaction of two surface proteins of *L. monocytogenes*, InlA and InlB, with their respective host receptors [2], [3], [4].

The Trw locus was laterally acquired during evolution of the bartonellae. It is present in the largest sub-branch of the genus tree, comprising 13 species that are adapted to diverse mammalian reservoir hosts, while it is absent from human-specific *B. bacilliformis*, cat-specific *Bartonella clarridgeiae* and the species of the ruminent-specific sub-branch, which all diverted early during evolution of the bartonellae [6]. Interestingly, the acquisition of Trw by the modern lineage correlates with the loss of flagella, which are know to represent a major pathogencity factor for the invasion of erythroctes by *B. bacilliformis* and probably other flagellated bartonellae [25]. The Trw system of *Bartonella* represents an interesting example of a pathogenesis-related T4SS that evolved rather recently by functional diversification of a laterally acquired bacterial conjugation system. The *trw* locus displays characteristic features of a pathogenicity island and shares extensive similarity with the *trw* locus of IncW broad-host range plasmid R388 encoding a genuine conjugation system. The *trw* loci of *Bartonella* and R388 are colinear, except for multiple tandem gene duplications of *trwL* and *trwJ*-*trwH* in *Bartonella*. Complementation of R388 derivatives carrying mutations in different *trw* genes with their *Bartonella* homologues allowed to demonstrate functional interchangability for some T4SS components [20], [26], underscoring the structural and functional conservation of individual subunits of these functionally diversified T4SSs. However, a major difference between these homologous systems is the lack of the coupling protein TrwB in *Bartonella*, which in R388 is required for export of T4SS substrates. The lack of TrwB in *Bartonella* thus indicates that its Trw system may not be capable of translocating substrates. However, the multiple copies of *trwL* and *trwJ* in the *Bartonella trw* locus encode variant forms of surface-exposed pilus components, which probably are all co-expressed [20], indicating that the primary function of the *Bartonella* Trw system may be the formation of variant pilus forms [25]. Based on the essential role of the Trw system for adhesion to erythrocyte and its role in determining host range it is conceivable to assume that these variant pili may facilitate the specific interaction with polymorphic erythrocyte receptors, either within the reservoir host population (e.g. different blood group antigens), or among different reservoir hosts. Phylogenetic analyses and calculation of the non-synonymous (*d*N) and synonymous (*d*S) substitution frequencies of different *trw* genes indeed demonstrated that *trwJ* and *trwL* homologs have diversified to much higher degree than other components of the Trw T4SS, within and among different species [23]. Together with the notion that the number of tandem repeats of *trwL* and *trwJIH* are variable among different *Bartonella* species these findings indicate that *trwL* and *trwJ* genes have been amplified and diversified several times during evolution. Horizontal transfer of such genes from a different bartonellae – similarly as we have demonstrated here for the entire *trw* operon of rat-specific *B. tribocorum* resulting in an extension of the host range of cat-specific *B. henselae* or human-specific *B. quintana* to rat – or alternatively pre-adaption of superfluous copies of *trwL* and *trwJ* may represent realistic molecular evolutionary scenarios for host shifts and thereby the evolution of pathogens with an altered host-specificity as it has happened repeatedly during the evolution of the bartonellae. Future studies should identify the nature of the erythrocyte receptors targeted by the Trw system and their specific interaction that facilitate host-specific erythrocyte infection.

## Materials and Methods

### Ethics statement

Animals were handled in strict accordance with good animal practice as defined by the relevant European (European standards of welfare for animals in research), national (Information and guidelines for animal experiments and alternative methods, Federal Veterinary Office of Switzerland) and/or local animal welfare bodies. Animal work performed at the Biozentrum of the University of Basel was approved by the Veterinary Office of the Canton Basel City on June 2003 (licence no. 1741), and animal work performed at the Ecole Nationale Vétérinaire d'Alfort (ENVA/AFSSA) was approved by the institute's ethics committee on September 2005.

### Bacterial strains and growth conditions

*B. alsatica* (IBS 382^T^, CIP 105477^T^) [27], *B. birtlesii* (IBS 135^T^, CIP 106691^T^) [28], *B. henselae* (Houston-1, ATCC 49882^T^), *B. quintana* (Fuller^T^, ATCC VR-358^T^), *B. tribocorum* (IBS 506^T^, CIP 105476^T^) [29], *B. vinsonii subsp. berkhoffii* (ATCC 51672^T^), *B. vinsonii subsp arupensis* (ATCC 700727) [30] were grown for 5 days on Columbia agar containing 5% defibrinated sheep blood (CBA) in a humidified atmosphere with 5% C0_2_ at 35°C.

### Construction of bacterial strains

*B. tribocorum*-*gfp* containing a chromosomally-integrated *gfp*-expression cassette [8] was used as GFP-expressing *B. tribocorum* strain. GFP-expressing bacteria of other *Bartonella* species were obtained by electroporation with plasmid pJMBGFP as previously described [31], [32]. This plasmid was extracted and purified from *B. quintana* using a Midi Prep Kit (Qiagen). The electroporation procedures was described previously [31]. Transformed bacteria were selected by plating on CBA-Km. A signature-tagged mutant library of *B. birtlesii* IBS135^T^ was constructed as described for *B. tribocorum* [6], [12]. Cosmid pAB2 encoding the entire *trw* locus of *B. tribocorum* [20], [33], [34] was introduced into *B*. *henselae* and *B. quintana* by three parental mating [34], [35].

### *In vitro* infection of erythrocytes

Erythrocytes from peripheral blood of mice (Balb/C), cats, rats (Wistar) and humans were isolated and purified by Ficoll gradient centrifugation. After washing in PBS, they were maintained in F12 modified medium [supplemented with 10% fetal calf serum, 2 mM glutamine, 1 mM sodium pyruvate, 0.1 mM Hepes, 257 mM histidine, 0.1 mg/ml hematin/histidine, non-essential amino acid (Gibco, FRANCE)] at 2×10^8^/ml. For *in vitro* infection experiments, *Bartonella* species were grown on CBA or CBA-km (*Bartonella*-*gfp* and STM mutants) plates. After 5 days of culture (10 days for GFP-expressing *Bartonella*), bacteria were harvested, washed, suspended in PBS, and added to erythrocytes at a multiplicity of infection (MOI, calculation based on 1 OD_600 nm_ = 3×10^9^ bacteria/ml) varying from 0.01 to 10 and incubated at 35°C in 5% C0_2_ for various periods of time (from 1 to 3 days).

### Detection of erythrocyte-associated bacteria

Colonization of erythrocytes by *Bartonella* was assessed and quantified by the gentamicin protection assay, flow cytometry and confocal microscopy. For the quantification of intracellular bacteria by gentamicin protection, 100 µl were withdrawn from the invasion mixtures after 1, 2 or 3 days of *in vitro* infection. Mouse erythrocytes were separated from non-associated bacteria by washing 3 times with PBS and centrifuged at 500 g for 5 min. Erythrocytes were then incubated for 2 h at 35°C with gentamicin sulfate (250 µg/ml) to kill residual extracellular bacteria. Erythrocytes were then washed three times in PBS to remove the antibiotic and intracellular bacteria were released from erythrocytes by hypotonic lyses of erythrocytes in 10 µl of sterile water by freezing at −20°C for 15 min. After thawing, serial dilutions of bacteria in PBS were inoculated onto CBA plates and incubated at 35°C for 5 days before being counted. For data presentation, all measurements were expressed as the number of CFU/10^10^ erythrocytes (corresponding to ≈1 ml of blood).

For flow cytometric detection of erythrocyte-associated with GFP-expressing bacteria, measurements were performed at day 1, 2, 3 after *in vitro* infection of erythrocytes with ten days old bacterial cultures. 100 µl of the infection mixtures was washed 3 times in PBS and fixed for 10 min with 0.8% paraformaldehyde and 0.025% glutaraldehyde. After fixing, erythrocytes were analyzed by flow cytometry (FACScan, Becton Dickinson Bioscience, France). For flow cytometric detection of erythrocytes associated with bacteria that do not express GFP (abacteremic mutants), measurements were performed at day 2 after *in vitro* infection. 100 µl of the infection mixtures was washed 3 times in PBS and fixed for 10 min with 0.8% paraformaldehyde and 0.025% glutaraldehyde. After fixing, erythroctes/mutants association was revealed with mouse anti-*B. birtlesii* serum and anti-mouse FITC antibodies (Santa Cruz Biotechnology, Santa Cruz, CA,USA) and analyzed by flow cytomtry. Data were analyzed using the CellQuestPro software, version 4.0.2. Data for 10′000 gated erythrocytes were collected and analyzed.

For confocal microscopy, 100 µl of the infection mixtures was washed three times in PBS and the erythrocytes cell surface was stained using goat anti-mouse GPA antibodies (Santa Cruz Biotechnology, California, USA) and labelled with anti- goat-PE-antibodies (ImmunoQuest Antibody, North Yorkshire, UK). Samples were viewed with a Nikon Eclipse C1 Plus confocal laser scanning microscope (Nikon, Amstelveen, Netherlands) with detection in channel 1 (GFP fluorescence) and channel 2 (PE fluorescence) at original magnification x100.

### STM library

The transposon vectors pHS006-Tag-001 to pHS006-Tag-036 each contained an *oriT* for conjugative transfer, the *Himar1* transposon, a kanamycin resistant marker, a hyperactive transposase and one of 36 distinct signature-tags [6]. These 36 signature-tagged mariner transposon vectors were separately transferred from *E. coli* β2155 to *B. birtlesii* by two-parental mating as previously described [35]. From each mating, 96 single kanamycin-resistant *B. birtlesii* transconjugants were transferred to a 96-well plate with cryo-medium and stored at −80°C.

### Mouse infections

Eight weeks old female Balb/C mice from Charles River Laboratories were housed in an animal facility (2 animals/cage) and allowed to acclimate to the facility and the diet for at least 5 days prior infection. Food and water were provided *ad libitum*. 36 differently signature-tagged mutants were grown separately from the transposon library for each input pool. They were pooled in PBS immediately before infection, and used to infect two mice with a total inoculum of 5×10^7^ colony forming units (10 µl of OD_595_ = 1) in the ear dermis of Balb/C mice. The remainder of the input pools was heated at 100°C for 10 min and used as template for PCR detection. Fifty µl of blood were taken from the tail vein of the infected mice when bacteremia is peaking (days 7 and 14 post-infection) [9]. Bacteria released from erythrocytes by a freeze/thaw cycle were plated on CBA-km. After 10 days, bacterial colonies (output pool) were counted, harvested in PBS, suspended to OD_595_ = 1 and heated at 100°C for 10 min to be used as template for PCR detection. The rescreen was done following the same protocol using pools of nine mutants (two abacteremic mutants and seven mutants displaying a wild-type phenotype).

### PCR detection of abacteremic mutants

For signature-tag identification, the generic primer Srev01 corresponding to a sequence in the transposon and a set of tag-specific primer were used for amplification of a fragment of approximately 600 bp [6]. The conditions for the PCR were as follows: a first denaturation step at 95°C for 5 min, followed by 30 cycles of PCR with denaturation at 95°C for 1 min, annealing for 30 s at 52°C, and extension at 72°C for 1 min. The program was completed by an extension step at 72°C for 5 min. The amplified fragments were displayed on a 1% agarose gel. Mutants that were detected in the input pools and absent from the out put pools (days 7 and 14) in both mice were considered as abacteremic mutants.

### Identification and analysis of transposon insertion sites

Genomic DNA from abacteremic mutants, regrown from the library, was prepared with the ROCHE Genomic DNA Isolation Kit. Genomic DNA was sent to QIAGEN for sequencing with primers Tn_start_ and Tn_end_ [6]. The sequences obtained by the genomic sequencing were compared by BlastN to the nr data base of NCBI (http://blast.ncbi.nlm.nih.gov/Blast.cgi). The exact transposon insertion sites were found by comparing the genomic sequences to contigs of the ongoing *B. birtlesii* genome sequencing project by BlastN.

### Screening of abacteremic mutants for their capacity to infect murine erythrocytes

Mutants displaying an abacteremic phenotype were tested for their capacity to invade murine erythrocytes using the gentamicin protection assay. Each mutant was tested at MOI = 1 in at least two independent experiments performed in triplicate samples. For mutants displaying an impaired erythrocyte invasion phenotype, invasion assays were performed at least three times in triplicate samples and adhesion assays were tested at day 2 post infection by flow cytometric detection once in triplicate samples.

### Statistical analysis

Numerical data are reported as the mean of at least 3 replicate samples +/- standard errors of the means. Statistical significance of the data was measured by use of Student's t test. A p-value <0.05 was considered significant.

### Phylogenetic and evolutionary analysis

The sequence of the *B. birtlesii trw* locus was deposited under the EMBL-EBI accession no. FN555106. Sequence alignments were calculated with ClustalW as implemented in MEGA4. Phylogenetic trees were inferred by maximum likelihood methods with Paup 4.0 [36] and 100 bootstrap replicates were calculated. To select an appropriate substitution model the Akaike information criterion of Modeltest 3.7 was used [37]. The models obtained were general time reversible (GTR) + I for *trwFED* and *trwN*, transversion model (TVM) + I for *trwI*, and TVM + I + G for *trwJ* and *trwL*. Nonsynonymous (dN) and synonymous (dS) substitution frequencies were calculated using the method of Yang and Nielson [38] as implemented in the PAML package [39], [40].

## Supporting Information

Figure S1 Efficiency of *in vitro* adhesion of murine erythrocytes to *Bartonella* sp. Freshly isolated murine erythrocytes were infected with *Bartonella* sp.-GFP (MOI = 1, detection at two DPI). The percentage of erythrocytes associated with bacteria were quantified by flow cytometric analysis. Representative data for the fluorescence (FL-1) of 10'000 erythrocytes are shown as histogram plots.
(0.08 MB TIF)

Figure S2 Efficiency of in *vitro* adhesion of murine erythrocytes to abacteremic mutants. Freshly isolated murine erythrocytes were infected with *B. birtlesii* abacteremic mutants (MOI = 1, detection 2 DPI). Association between erythrocytes and bacteria was revealed with mouse anti-*B. birtlesii* polyclonal serum and labelled with anti-mouse FITC antibody. The percentage of erythrocyte associated with bacteria was quantified by flow cytometric analysis. Representative data for the fluorescence (FL-1) of 10'000 erythrocytes are shown as histogram plots.
(0.20 MB TIF)

Figure S3 Genetic organization of the *Bartonella trw* locus, and phylogenies and synonymous (*d*S) vs. nonsynonymous (*d*N) substitution frequencies of the encoded trw genes. (A) Gene order structure of the trw locus of *B. birtlesii* and comparison to other *Bartonella* species. The copy number of amplified genes or segments in other *Bartonella* species is indicated within brackets. Maximum Likelihood phylogenies of (B) the concatenated nucleotide alignments of *trwF, trwE,* and *trwD*, the nucleotide alignments of (C) *trwJ* copies, (D) *trwI*, (E) *trwL* copies, and (F) *trwN* of *B. birtlesii (Bb), B. grahamii (Bg), B. henselae (Bh), B. quintana (Bq)*, and *B. tribocorum (Bt)*. For *trwJ* (C) and *trwL* (E), the range of pairwise *d*N/*d*S ratios of different phylogenetic subclusters (shaded areas) are indicated at the upper right of each cluster. For *trwL1*, the range of pairwise *d*N/*d*S ratios is indicated as well, although they do not cluster. (G) The pairwise *d*N/*d*S ratios of orthologous trw genes and the two adjacent genes *ubiH* and *sdhA* of *B. birtlesii* and *B. grahamii, B. henselae, B. quintana*, or *B. tribocorum* are plotted according to their gene order. For the tandem repeated genes *trwL, trwJ, trwI*, and *trwH* only *trwL5, trwJ1, trwI1*, and *trwH1* are shown, since ortholog assignment is difficult for the others due to copy number variation and the occurrence of recombination among different species [23].
(0.46 MB JPG)

Table S1 Genotypic characterization of abacteremic mutants of *B. birtlesii* obtained by signature-tagged mutagenesis (STM). The columns BARBAKC, BH, BQ and BT list the extensions of systematic names of orthologous genes from the published genomes of *B. bacilliformis* (accession no. CP000524), *B. henselae* (accession no. BX897699), *B. quintana* (accession no. 897700) and *B. tribocorum* (accession no. AM260525), respectively. * The *in vitro* erythrocate invasion phenotype of each mutant was determined by the gentamicin protection assay after 1 day of infection (triplicate samples) and categorized as normal (>70% of wild-type), reduced (<70% of wild-type but >1% of wild-type) or none (<1% of wild-type). Mutants with reduced or none in vitro invasion were tested again (n = 3) and the resulting mean and SD of all three experiments are represented in Figure 4.
(0.15 MB DOC)
