# Supplementary figures and images for: The Trw Type IV Secretion System of Bartonella Mediates Host-Specific Adhesion to Erythrocytes
Source: PLoS Pathog. 2010 Jun 10;6(6):e1000946. doi: 10.1371/journal.ppat.1000946 (PMC2883598; doi:10.1371/journal.ppat.1000946)

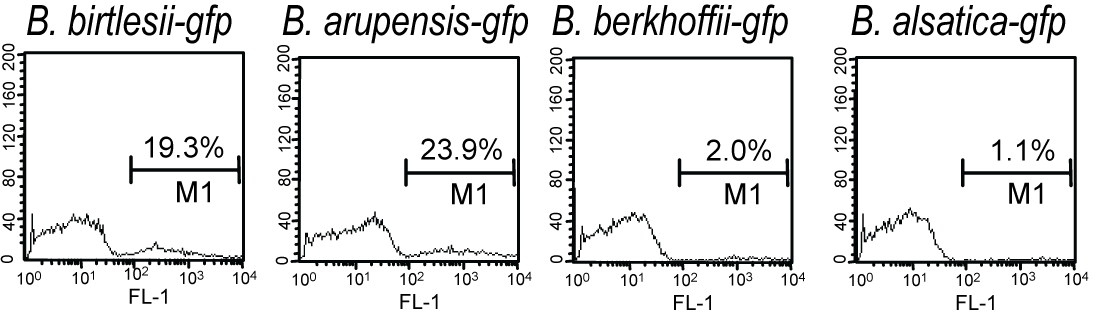

Supplement: Figure S1 — Efficiency of in vitro adhesion of murine erythrocytes to Bartonella sp. Freshly isolated murine erythrocytes were infected with Bartonella sp.-GFP (MOI = 1, detection at two DPI). The percentage of erythrocytes associated with bacteria were quantified by flow cytometric analysis. Representative data for the fluorescence (FL-1) of 10'000 erythrocytes are shown as histogram plots. (0.08 MB TIF) [file ppat.1000946.s001.tif]

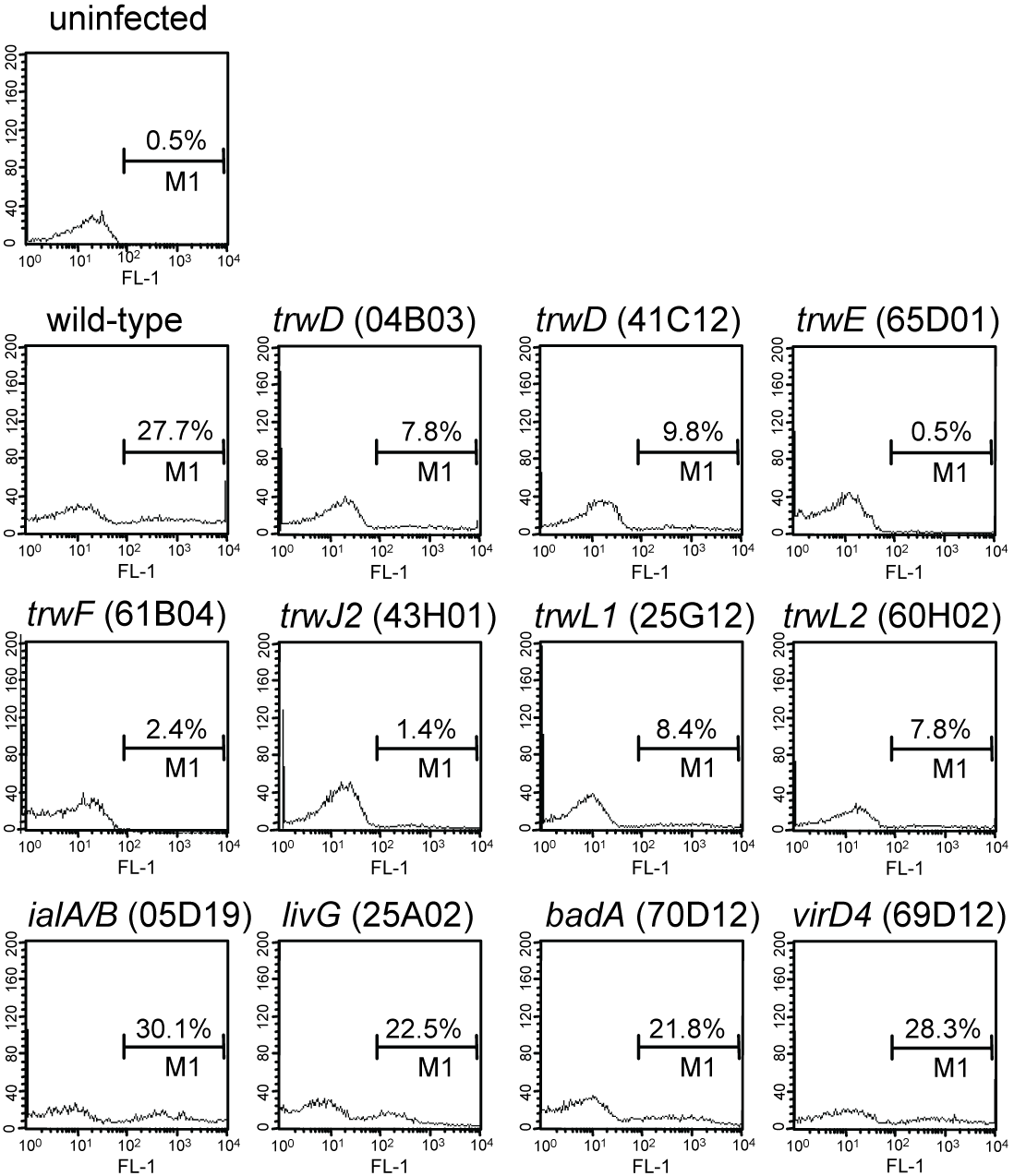

Supplement: Figure S2 — Efficiency of in vitro adhesion of murine erythrocytes to abacteremic mutants. Freshly isolated murine erythrocytes were infected with B. birtlesii abacteremic mutants (MOI = 1, detection 2 DPI). Association between erythrocytes and bacteria was revealed with mouse anti-B. birtlesii polyclonal serum and labelled with anti-mouse FITC antibody. The percentage of erythrocyte associated with bacteria was quantified by flow cytometric analysis. Representative data for the fluorescence (FL-1) of 10'000 erythrocytes are shown as histogram plots. (0.20 MB TIF) [file ppat.1000946.s002.tif]

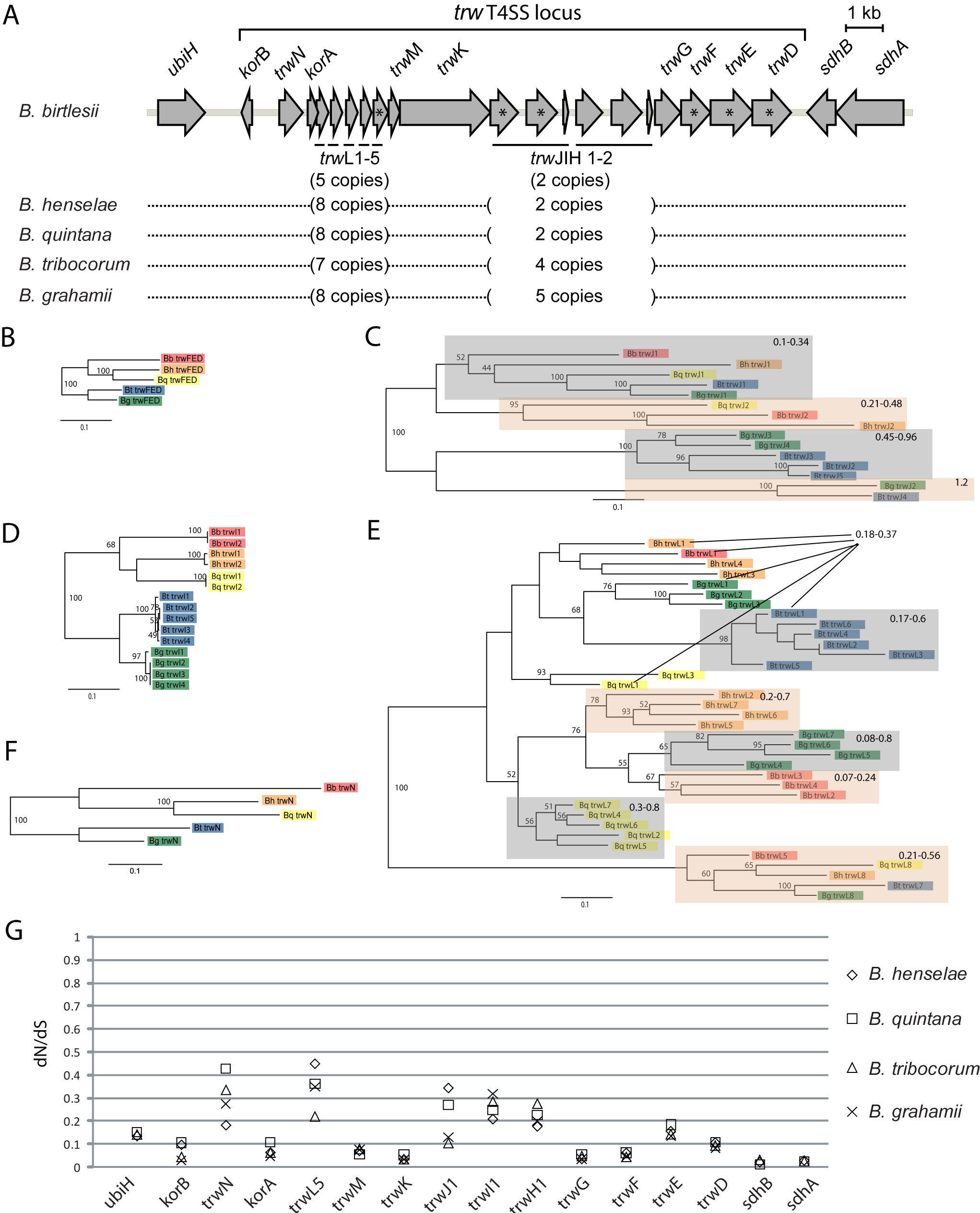

Supplement: Figure S3 — Genetic organization of the Bartonella trw locus, and phylogenies and synonymous (dS) vs. nonsynonymous (dN) substitution frequencies of the encoded trw genes. (A) Gene order structure of the trw locus of B. birtlesii and comparison to other Bartonella species. The copy number of amplified genes or segments in other Bartonella species is indicated within brackets. Maximum Likelihood phylogenies of (B) the concatenated nucleotide alignments of trwF, trwE, and trwD, the nucleotide alignments of (C) trwJ copies, (D) trwI, (E) trwL copies, and (F) trwN of B. birtlesii (Bb), B. grahamii (Bg), B. henselae (Bh), B. quintana (Bq), and B. tribocorum (Bt). For trwJ (C) and trwL (E), the range of pairwise dN/dS ratios of different phylogenetic subclusters (shaded areas) are indicated at the upper right of each cluster. For trwL1, the range of pairwise dN/dS ratios is indicated as well, although they do not cluster. (G) The pairwise dN/dS ratios of orthologous trw genes and the two adjacent genes ubiH and sdhA of B. birtlesii and B. grahamii, B. henselae, B. quintana, or B. tribocorum are plotted according to their gene order. For the tandem repeated genes trwL, trwJ, trwI, and trwH only trwL5, trwJ1, trwI1, and trwH1 are shown, since ortholog assignment is difficult for the others due to copy number variation and the occurrence of recombination among different species [23]. (0.46 MB JPG) [file ppat.1000946.s003.jpg]
