# Supplementary material for: The Trw Type IV Secretion System of Bartonella Mediates Host-Specific Adhesion to Erythrocytes
Source: PLoS Pathog. 2010 Jun 10;6(6):e1000946. doi: 10.1371/journal.ppat.1000946 (PMC2883598; doi:10.1371/journal.ppat.1000946)
Supplement: Table S1 — Genotypic characterization of abacteremic mutants of B. birtlesii obtained by signature-tagged mutagenesis (STM). The columns BARBAKC, BH, BQ and BT list the extensions of systematic names of orthologous genes from the published genomes of B. bacilliformis (accession no. CP000524), B. henselae (accession no. BX897699), B. quintana (accession no. 897700) and B. tribocorum (accession no. AM260525), respectively. * The in vitro erythrocate invasion phenotype of each mutant was determined by the gentamicin protection assay after 1 day of infection (triplicate samples) and categorized as normal (>70% of wild-type), reduced (<70% of wild-type but >1% of wild-type) or none (<1% of wild-type). Mutants with reduced or none in vitro invasion were tested again (n = 3) and the resulting mean and SD of all three experiments are represented in Figure 4. (0.15 MB DOC) [file ppat.1000946.s004.doc]

**Supporting Table S1:** **Genotypic characterization of abacteremic mutants of *B. birtlesii* obtained by signature-tagged mutagenesis (STM).** The columns BARBAKC, BH, BQ and BT list the extensions of systematic names of orthologous genes from the published genomes of *B. bacilliformis* (accession no. CP000524)*, B. henselae* (accession no. BX897699), *B. quintana* (accession no. 897700) and *B. tribocorum* (accession no. AM260525), respectively. * The *in vitro* erythrocate invasion phenotype of each mutant was determined by the gentamicin protection assay after 1 day of infection (triplicate samples) and categorized as normal (>70% of wild-type), reduced (<70% of wild-type but >1% of wild-type) or none (<1% of wild-type). Mutants with reduced or none *in vitro* invasion were tested again (n=3) and the resulting mean and SD of all three experiments are represented in Figure 4.

| **Gene Name** | **Mutant** | **Putative function** | **BARBAKC** | **BH** | **BQ** | **BT** | ***In vitro* infection*** |
| --- | --- | --- | --- | --- | --- | --- | --- |
| **Adhesion/Invasion** |  |  |  |  |  |  |  |
| *badA* | 05A04 | adhesin | 583_0314 | 01490  01510 | 01390  01400  01410 | 0168 | normal |
| *badA* | 70D12 | adhesin | 583_0314 | 01490  01510 | 01390  01400  01410 | 0168 | normal |
| *ialA/ialB* | 05D10 | invasion associated gene B | 583_0326 | 01650 | 01550 | 0181 | reduced |
| *ibaA* | 44H12 | putative inducible autotransporter | 583_1132 | 13160 | 10410 | 1655 | normal |
| *omp43* | 43G09 | outer membrane protein, adhesin | 583_0447 | 12500 | 09890 | 1902 | normal |
| *trwD* | 04B03 | T4SS component, VirB11 homolog | - | 15760 | 12680 | 2533 | reduced |
| *trwD* | 41C12 | T4SS component, VirB11 homolog | - | 15760 | 12680 | 2533 | reduced |
| *trwE* | 65D01 | T4SS component, VirB10 homolog | - | 15750 | 12670 | 2532 | none |
| *trwF* | 61B04 | T4SS component, VirB9 homolog | - | 15740 | 12660 | 2531 | none |
| *trwJ2* | 43H01 | T4SS component, VirB5 homolog | - | 15670  15700 | 12590  12620 | 2519  2522  2524  2526  2528 | none |
| *trwL1* | 25G12 | T4SS component, VirB2 homolog | - | 15570  15580  15590  12600  12610  12620  12630  12640 | 12490  12500  12510  12520  12530  12540  12550  12560 | 2511  2512  2513  2514  2515  2516  2516a | none |
| *trwL2* | 60H02 | T4SS component, VirB2 homolog | - | 15570  15580  15590  12600  12610  12620  12630  12640 | 12490  12500  12510  12520  12530  12540  12550  12560 | 2511  2512  2513  2514  2515  2516  2516a | none |
| *virB4* | 61H02 | T4SS component, VirB4 homolog | - | 13280 | 10550 | 1691 | normal |
| *virD4* | 69D12 | T4SS component, VirD4 homolog | - | 13380 | 10640 | 1701 | normal |
| *virD4* | 79C12 | T4SS component, VirD4 homolog | - | 13380 | 10640 | 1701 | normal |
|  | 61C01 | autotransporter protein | - | 13030 | 10290 | 1796 | normal |
| **Iron uptake** |  |  |  |  |  |  |  |
| *hutA* | 5B10 | outer membrane heme receptor | 583_0460 | 04970 | 04160 | 0774 | normal |
| **Transport function** |  |  |  |  |  |  |  |
| *ilvE* | 45B03 | amino acid transporter | 583_0747 | 10010 | 07730 | 1376 | normal |
| *livF* | 04A08 | amino acid transporter | - | 08250 | 06330 | 1144 | normal |
| *livG* | 25A02 | amino acid transporter | - | 08260 | 06320 | 1145 | reduced |
| *livH* | 45C07 | ABC transporter | - | 08280 | 06300 | 1147 | normal |
| *phaA* | 05G09 | K+/H+ transmembrane protein | 583_0030 | 16460 | 13360 | 2670 | normal |
| **Cell stress response** |  |  |  |  |  |  |  |
| *ibpA* (*hsp20*) | 86B07 | chaperon | 583_0614 | 07300 | 05230 | 1333 | normal |
| *hslO* (*hsp33*) | 41A03 | chaperon | 583_1292 | 01080 | 00990 | 0118 | normal |
| **Metabolism / cell integrity** |  |  |  |  |  |  |  |
| *carD* | 41C07 | transcriptional regulator factor | 583_0123 | 15240 | 12150 | 2444 | normal |
| *glnE* | 70D02 | glutamate ammonialigase adenyl  transferase | - | 4800 | 04000 | 0707 | normal |
| *ftsK* | 15G10 | cell division transmembrane protein | 583_0291 | 03840 | 02850 | 0572 | normal |
| *cobS* | 44G10 | cobalamin biosynthesis | 583_0080 | 15880 | 12800 | 2554 | normal |
| *lpcC* | 69H08 | lipopolysaccharide core biosynthesis mannosyltransferase | 583_0983 | 11690 | 09300 | 0746 | normal |
| *mfd* | 41B10 | transcription repair coupling factor | 583_0798 | 08750 | 05840 | 1197 | normal |
| **Unknown function** |  |  |  |  |  |  |  |
|  | 43H05 | unknown function | - | 03150 | - | 0332  0505  1229  1275  1394  1812  2614 | normal |
| BA0981 | 65D04 | putative exported protein | - | 02590 | 02450 | 0286 | normal |
| BA1484 | 15A08 | putativemembrane protein | 583_1009 | 11960 | 09380 | 0713 | normal |
| BA1559 | 35D02 | helicase/methyltransferase | - | 15450 | - | 0164  0455  0541  1006  1021  1035  1053  1080  1105  2491 | normal |
| BA1819 | 41C02 | unknown function | - | 09350 | - | 0466  1089  1090  2281  2282 | normal |
| BA1566 | 61D04 | unknown function | - | - | - | 1926 | normal |
|  | 05H01 | conserved/putative (Tm helices) membrane protein | 583_0492 | 05300 | 04480 | 0812 | normal |
|  | 44G12 | putative efflux transport protein | - | 12560 | - | 1909 | normal |
| **Phage origin** |  |  |  |  |  |  |  |
| BA1301 | 86C10 | putative anti-repressor protein | 583_1070 | 06900  02990  03430  03240  03670  03690 | - | 0325  0355  0372  0373  0431  0470  0486  0494  0556  0557  0954  0976  2290  2301 | normal |
| BA1052 | 60B07 | Putative anti-repressor protein | - | 02890  03020  03250  03440  0345  03460  03470 | - | 0475 | normal |
| **Intergenic region** |  |  |  |  |  |  |  |
|  | 83D04 | ig | 583_1301/  583_1302 | 00970/00960 | 00900/0089 | tRNA-BT0001/BT0082 | normal |
|  | 69C09 | ig, close to putative transcriptional regulator | 583_1248 | 1359014160  14370  14380  14970 | 02090 | 2389  2390  2397  2399  2400 | normal |
|  | 04A01 | ig | 583_1132 | 13140 or 13160 | 10380 | 1660  or  1661 | normal |
|  | 69B07 | ig | 583_0094/  583_0093 | 15500/15510 | 12420/ 12430 | 2497/  2504 | normal |
|  | 86C05 | ig | 583_1019/  583_1020 | 12050/12060 | 09460/ 09470 | 1641/ 1642 | normal |
